# Use of sedative pharmacological agents among biomedical students during the coronavirus disease 2019 pandemic: a cross-sectional pilot study

**DOI:** 10.3325/cmj.2022.63.570

**Published:** 2022-12

**Authors:** Monika Burja Vladić, Magdalena Jelkić, Marijan Mandić, Kristina Peroš

**Affiliations:** 1 School of Dental Medicine, University of Zagreb, Croatia; 2 University of Zagreb, Faculty of Economics and Business, Zagreb, Croatia; 3 Depertment of Pharmacology, University of Zagreb, School of Dental Medicine, Zagreb, Croatia; The first two authors contributed equally.

## Abstract

**Aim:**

To assess the use of sedative pharmacological agents (SPA) among students in Croatia during the coronavirus disease 2019 pandemic.

**Methods:**

An online questionnaire, specially developed for the research purposes, was completed by 1403 students. The questionnaire inquired about general characteristics, the effect of the pandemic and earthquakes on students’ lives, and SPA use (method of purchasing, modes, and frequency).

**Results:**

In the total sample, the SPA use before the pandemic significantly correlated with that after the beginning of the pandemic (*P* < 0.001). After the beginning of the pandemic, medical students used significantly more SPA than other biomedical students (*P* = 0.017). When compared with non-biomedical students, biomedical students did not differ significantly in SPA use after the beginning of the pandemic (*P* = 0.365).

**Conclusion:**

Medical students used more SPA than other biomedical students. Biomedical students did not differ in SPA use from non-biomedical students.

The coronavirus disease 2019 (COVID-19) pandemic (1) has increased the number of mental health problems in the general population, in particular among health professionals, patients suffering from chronic non-infectious diseases, patients with COVID-19, and self-isolating persons (2). Healthcare professionals, who are generally prone to burnout and depressive symptoms (3,4), faced sudden changes and increased workload, which has already been documented during the SARS epidemic (5). Healthcare professionals working in high-risk sites had two-three times higher risk of developing posttraumatic stress syndrome compared with those who were not exposed (6). Another group affected by the pandemic are students due to the increased feeling of depression and anxiety (7-13). A high percentage of students (71.26%) in the United States suffered from depression and anxiety, and 18% of them had suicidal thoughts (7). However, other studies found that medical students, although they already have an increased risk of mental disorders compared with the general population, did not suffer additional psychological consequences of the pandemic (3,14,15). There is a dearth of research documenting the details associated with the use of sedative pharmacological agents (SPA), prescription or over-the-counter, in the student population during the COVID-19 pandemic.

In 2020, the wider Zagreb area was hit by a series of earthquakes (the strongest occurring on March 21, 2020, and December 29, 2020), which might have additionally affected the mental health of the student population. The aim of this study was to assess the SPA use among biomedical students at higher education institutions of Croatia, before and after the beginning of the COVID-19 pandemic. We tested two null hypotheses: 1) there is no significant difference in the prevalence of SPA use between biomedical students and non-biomedical students after the beginning of the pandemic and 2) there is no significant difference in the prevalence of SPA use among subgroups of biomedical students and the group of non-biomedical students after the beginning of the pandemic.

## Methods

The study involved students who were enrolled in full-time or part-time undergraduate, graduate, or integrated undergraduate and graduate studies in all higher-education institutions in Croatia in the 2020/2021 academic year. Overall, 1436 students filled out the questionnaire. Postgraduate doctoral students and students over the age of 30 were excluded. This criterion was applied to obtain a more homogenized, representative sample of respondents with similar life circumstances. The final sample involved 1403 students (62.4% women). Participation in the survey was voluntary and anonymous. The research was approved by the Ethics Committee of the School of Dental Medicine, University of Zagreb.

**Figure 1:**
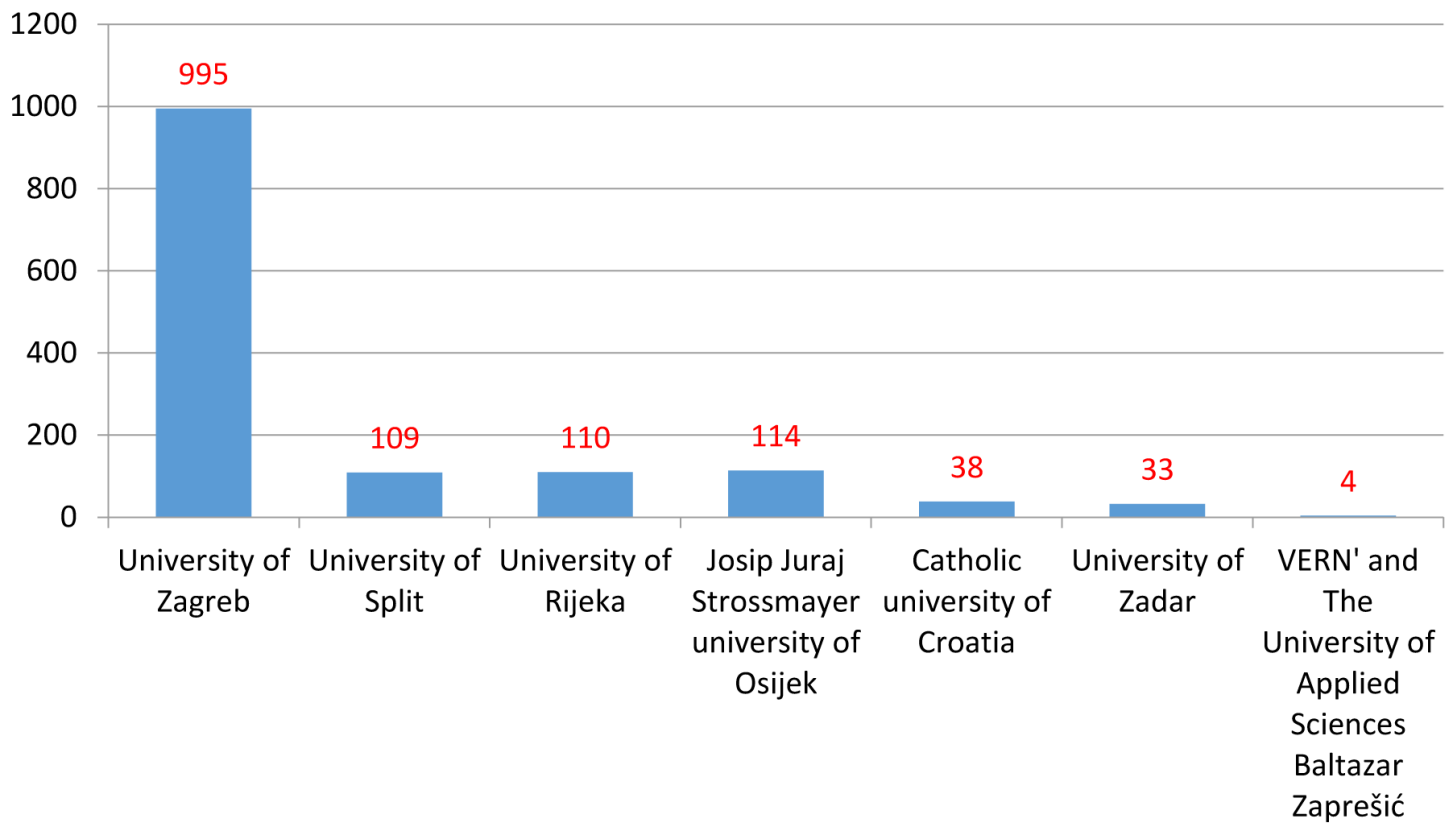
The structure of respondents according to higher education institutions in Croatia.

A questionnaire, designed for this purpose, was created in Google Forms. The collection of responses began in mid-December 2020 and ended in mid-April 2021. The survey was distributed via email addresses and social networks (Facebook and WhatsApp). The detailed structure of respondents according to the higher education institution is shown in Figure 1.

**Figure 2:**
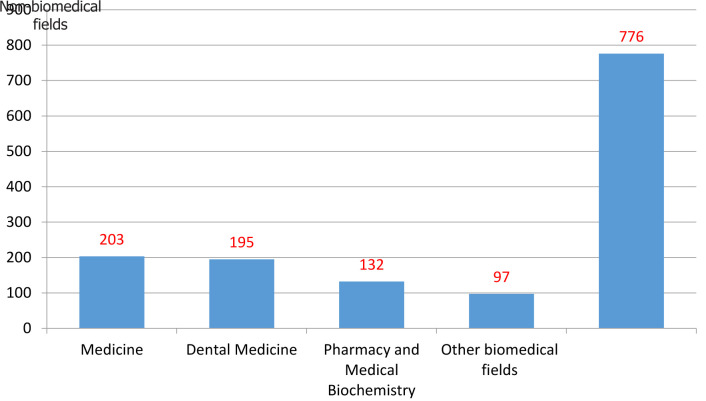
The structure of respondents according to the enrolled academic program.

The respondents were classified as biomedical (44.7%) or non-biomedical students (55.3%). Biomedical students were classified into four subgroups: 1) medicine, 2) dental medicine, 3) pharmacy, and 4) medical biochemistry and other biomedical fields, including veterinary medicine, nursing, midwifery, clinical nutrition, sanitary engineering, medical laboratory diagnostics, physiotherapy, biotechnology, and drug research. The structure of respondents according to the enrolled academic programs is shown in Figure 2.

A total of 10.9% of respondents had chronic diseases, 4.9% of respondents had psychiatric disorders, and 26.5% of respondents were smokers.

### Questionnaire

The questionnaire consisted of 35 questions (Supplementary material(Supplementary Questionnaire)). It inquired about general demographic data, the impact of earthquakes and the pandemic on students’ lives, and the use of over-the-counter and prescription SPA after the beginning of the pandemic. Two groups of questions inquired about the use of most common prescription SPA, including tranquilizers, anxiolytics, hypnotics, and antidepressants. The brand names were listed, and the respondents were allowed to add answers that were not offered. The questions were mostly multiple choice, and several were check-box, Likert-type, or required short answers.

### Statistical analysis

Normality of distribution was tested with the Shapiro-Wilk test. Data on general characteristics of the respondents, the impact of the pandemic and earthquake on students’ lives, and on the method of purchasing, modes, and frequency of SPA use were summarized with descriptive statistics. The total use of SPA before and after the beginning of the pandemic was compared with the χ^2^ test. The between-group testing was performed with the *t* test. Post-hoc Tukey test was used. Since this is a large sample (n = 1403), a certain asymmetry of the distributions expressed by skewness and kurtosis coefficients did not affect the validity of the results (16). The significance level was set at alpha = 0.05. Statistical analysis was performed with SPSS, version 25 (IBM Corp, Armonk, USA).

## RESULTS

SPA were used by 15.8% of respondents before the pandemic, and by 13% after the beginning of the pandemic.

In the total sample, the SPA use before the pandemic significantly correlated with the SPA use after the beginning of the pandemic (*P* < 0.001) (Table 1). Most respondents who used SPA before the pandemic continued to use them after the beginning of the pandemic. The odds of not taking SPA in the entire sample was 39.019 times the odds of taking SPA, both before and after the beginning of the pandemic (Table 1).

**Table 1:** The association of sedative pharmacological agents (SPA) use before and after the beginning of SARs-CoV-2 pandemic in the entire student population*

Frequencies			Have you ever taken SPA after the beginning of the SARS-CoV-2 pandemic?					
		yes		no	total	Odds ratio	95% confidence interval	
**Have you ever taken SPA before the beginning the SARS-CoV-2 pandemic?**	yes	**136**		**86**	222	39.019	26.161	58.196
	no	**46**		**1135**	1181			

*Pearson Chi-Square P value <0.001.

Both before and after the beginning of the pandemic, there was no difference between biomedical and non-biomedical students in SPA use (Table 2, *P* = 0.577, *P* = 0.365).

**Table 2:** The use of sedative pharmacological agents (SPA) in biomedical and non-biomedical students before and after the beginning of the SARS-CoV-2 pandemic

	Yes	No	Total	Odds ratio	95% confidence interval		
**Have you ever taken SPA before the SARS-CoV-2 pandemic?**							
Biomedical students	**103**	**524**	627	1.085	0.814*		1.447*
Non-biomedical students	**119**	**657**	776				
**Have you ever taken SPA after the beginning of the SARS-CoV-2 pandemic?**							
Biomedical students	**87**	**540**	627	1.155	0.845^†^	1.578^†^	
Non-biomedical students	**95**	**681**	776				

*Pearson Chi-Square P value: 0.577.

†Pearson Chi-Square P value: 0.365.

Among biomedical students, the consumption of SPA was lower than expected among students of dental medicine and pharmacy and medical biochemistry before and after the beginning of the pandemic, but it was higher than expected among students of medicine and other biomedical fields (*P* = 0.006, *P* = 0.017). Biomedical students observed together did not differ significantly from non-biomedical students in terms of SPA use before and after the beginning of the pandemic (Table 3). Medical students had higher SPA use than other subgroups (Supplementary Table 1 (Supplementary Table 1)and Supplementary Table 2(Supplementary Table 2)).

**Table 3:** The use of sedative pharmacological agents (SPA) in non-biomedical students and subgroups of biomedical students before and after the beginning of the pandemic

Have you ever taken SPA before the beginning of the SARS-CoV-2 pandemic?*			Yes	No	Total
**Subgroups of biomedical students**	medicine		47	156	203
	dental medicine		22	173	195
	pharmacy and medical biochemistry		15	117	132
	other biomedical fields		19	78	97
**Non-biomedical students**	other programs		119	657	776
**Have you ever taken SPA after the beginning of the SARS-CoV-2 pandemic†**					
**Subgroups of bio-medical students**	medicine	40		163	203
	dental medicine	19		176	195
	pharmacy and medical biochemistry	13		119	132
	other biomedical fields	15		82	97
**Non-bio-medical students**	other programs	95		681	776

*Pearson Chi-Square P value: 0.006.

†Pearson Chi-Square P value: 0.006.

Most of the respondents used SPA without prescription before and after the beginning of the pandemic. Furthermore, most of the respondents used SPA symptomatically or as needed, several times a week (Supplementary Table 3(Supplementary Table 3), Supplementary Table 4(Supplementary Table 4), and Table 4). The most commonly used SPA before and after the beginning of the pandemic was diazepam (Table 5). Among 1403 respondents, 74 respondents used SPA only when needed and 105 continued to take then as needed. Overall, 152 respondents were aware of harmful consequences of long-term SPA intake. A small number of students stated that they were aware of the dangers of long-term substance use but did not pay attention, while a negligible number of respondents stated they were not aware of the dangers (Table 6, Table 7).

**Table 4:** Number (%) of students taking sedative pharmacological agents (SPA) before and after the beginning of the pandemic

Frequency of taking SPA	Before	After
Daily	33 (2.4)	32 (2.3)
Up to 5 times a week	20 (1.4)	26 (1.9)
Up to 5 times a month	30 (2.1)	41 (2.9)
Several times a year	139 (9.9)	83 (5.9)
Did not use	1181 (84.2)	1221 (87.0)

**Table 5:** Commonly used sedative pharmacological agents (SPA) before and after the beginning of the pandemic

SPA	Before	After
Alprazolam	77 (5.5)	70 (5.0)
Bromazepam	17 (1.2)	12 (0.9)
Diazepam	114 (8.1)	91 (6.5)
Oxazepam	10 (0.7)	8 (0.6)
Lorazepam	4 (0.3)	3 (0.2)
Midazolam	1 (0.1)	1 (0.1)
Nitrazepam	1 (0.1)	2 (0.1)
Flurazepam	2 (0.1)	1 (0.1)
Antidepressants	25 (1.8)	25 (1.8)
Persen	20 (1.4)	9 (0.6)
Others	41 (2.9)	37 (2.6)

**Table 6:** Respondents who stopped taking and who continued to take sedative pharmacological agents (SPA) after the beginning of the pandemic

Discontinuation of medication or continued use?	Absolute frequencies	%
Used it only when needed and don't take it anymore	74	5.3
Still taking it as needed	105	7.5
Used it regularly for a few weeks	3	2
Did not use	1221	87.0
Total	1403	100.0

**Table 7:** Awareness of the respondents about long-term and irrational use of sedative pharmacological agents (SPA)

Are you aware of the harmfulness of long and irrational use of these drugs?	Absolute frequencies	%
Yes, and I use them carefully.	152	10.8
Yes, but don't care so much about it.	26	1.9
Unaware	4	3
Did not use	1221	87.0
Total	1403	100.0

At the time of the March 22, 2020 earthquake, 46.2% of respondents were in or around Zagreb.

Three respondents (0.2%) were hospitalized due to SARS-CoV-2 infection. In general, students were concerned about the possible impact of the virus on the health of their loved ones and about the economic consequences of the pandemic. On the other hand, students did not believe that earthquake significantly affected their life and that the pandemic and earthquake significantly changed their sources of funding. More non-biomedical students said they were concerned about how the pandemic would affect their future employment. On the other hand, significantly more biomedical students were concerned that adapting schooling to pandemic circumstances would affect their competence in the future workplace. Another concern among biomedical students was related to the discontinuation of practical classes in the summer semester of the 2019/2020 academic year and the reduction in the number practical classes in the 2020/2021 academic year.

Female respondents found it more difficult to adapt to online learning and felt more technical difficulties in following online classes. Female students also expressed significantly stronger concerns regarding reports on the number of infected and dead during the pandemic, the end of the academic year, future work competencies and employment, and how the pandemic and earthquake reduced the quality of their studying. Regardless of the difficulties, the majority of female students had other aggravating circumstances that affected their health. (Supplementary Table 5(Supplementary Table 5), Supplementary Table 6(Supplementary Table 6), Supplementary Table 7(Supplementary Table 7)).

## DISCUSSION

This study showed no difference in the SPA use in the total sample before and after the beginning of the pandemic. Other research showed an increase in the use of SPA after chronic stressors such as pandemics and acute stressors such as earthquakes (17-19). The lack of increase in the use of these substances may speak in favor of the psychological resilience of our students, a safer social environment that amortized the stress, or the presence of protective factors. Further research is needed to reveal elements that prevented the expected increase in the use of SPA.

This study found no significant difference in SPA use between biomedical students and non-biomedical students before and after the beginning of pandemic. The first null hypothesis was thus confirmed. Medical students were taking more SPA compared with non-biomedical students, but also compared with students of other biomedical fields such as dental medicine and pharmacy. The second null hypothesis was thus refuted.

SPA use among medical students has already been recognized. According to a study from 2000, 33% of students at the School of Medicine, University of Zagreb, were taking SPA due to anxiety, depression, stress, and insomnia (20). Furthermore, the prevalence of drug abuse among physicians was higher than in the general population. Further research is needed to elucidate the cause of these problems.

Our research showed that both before and after the beginning of the pandemic, 9.8% and 7.7% students, respectively, obtained SPA without prescription, Our results are in line with some other studies (21,22). At the same time, Abbasi-Ghahramanloo et al (23) found the rate to be higher (16.1%). In our study, SPA were taken symptomatically by most students, similar to the study by Schulenberg et al (24).

According to our results, after the beginning of the pandemic most students continued to use SPA, while a slightly smaller number stopped using them. An encouraging finding is that that the majority of students who used SPA were aware of the harmfulness of long and irrational SPA use and used SPA carefully. This is consistent with our results showing that most students were taking SPA symptomatically or as prescribed. Contrary to our findings, Grant et al (25) reported SPA abuse, defined as the SPA use without prescription, among students.

Of the other psychoactive substances, the largest number of students consumed marijuana. This is in line with other research (24) finding marijuana use to be common in a population aged 19 to 30 years.

Concerns expressed by our respondents about the effects of the pandemic and earthquakes on the quality of their studying and the end of the academic year, future work competencies, and employment are consistent with the results of Adam et al (26). However, while Adam et al did not find any gender differences in this respect, we observed significant gender differences, consistent with the results of other research (27-29). The fact that more female (62.4%) than male respondents (37.6%) participated in this survey should also be considered. The share of students in our research coincides with the total share of female students (62%) in Croatia in 2020/2021 (30). Further research is needed to determine the cause of these gender differences. When it comes to age structure, we cannot determine whether our sample is representative of the entire student population due to a lack of such data at the time of the study. Considering the type of study program, the division of the students into biomedical and non-biomedical fields was not the subject of a more detailed analysis.

In our study, significantly more biomedical students compared with non-biomedical students were concerned about the reports on the number of the COVID-19-infected and dead. The reason for this could be the knowledge and understanding of the topics related to the COVID-19 pandemic (31-36). Significantly more biomedical students were concerned that adapting schooling to the pandemic circumstances would affect their competence in the future workplace. This finding could be interpreted in the light of the current lack of medical staff throughout Europe, which was also emphasized during the pandemic. Similarly, in another study, students of School of Dental Medicine, University of Zagreb were worried about their competence in the future workplace because during the pandemic they had a limited opportunity to acquire clinical skills (26).

The study suffers from several limitations. To our knowledge, there is no standardized questionnaire on the use of SPA, so we created our own questionnaire. The investigation of the use of alcohol, marijuana, cocaine, heroin, and other drugs was not within the scope of this study. Further research is needed to assess the use of these agents alone or concomitantly with SPA. The number of participating students is not representative of the number of students of each university school. Given that the study was conducted during the COVID-19 pandemic, the only mode of distribution was online distribution, which could have reduced the interest among students.

Despite these limitations, this study provides a basis for further research on a larger number of respondents with more variables in order to elucidate the association between SPA use and anxiety during the pandemic and following earthquakes.

## Additional Material

Supplementary Questionnaire
