# Supplementary material for: Use of sedative pharmacological agents among biomedical students during the coronavirus disease 2019 pandemic: a cross-sectional pilot study
Source: Croat Med J. 2022 Dec;63(6):570–7. doi: 10.3325/cmj.2022.63.570 (PMC9837717; doi:10.3325/cmj.2022.63.570)
Supplement: Supplementary Questionnaire [file CroatMedJ_63_s001.pdf]

Supplementary questionnaire

The following questions refer to your demographic information:

1. Sex

a) Male

b) Female

2. Age - enter years numerically ¬____________________

3. Place of residence (selection of counties)

a) Zagrebačka

b) Krapina-Zagorska

c) Sisak-Moslavina

d) Karlovačka

e) Varaždinska

f) Koprivnica-Križevačka

g) Bjelovar-Bilogorska

h) Primorsko-Goranska

i) Lika-Senjska

j) Virovitica-Podravska

k) Požega-Slavonia

l) Brodsko-Posavina

m) Zadar

n) Osijek-Baranja

o) Šibenik-Knin

p) Vukovar-Srijemska

q) Split-Dalmatia

r) Istria

s) Dubrovnik-Neretva

t) Međimurska

u) City of Zagreb

4. At which University do you study?

a) University of Zagreb

b) University of Rijeka

c) University of Split

d) University in Osijek

5. Field of study:

a) medicine

b) dental medicine

c) veterinary medicine

d) nursing

e) midwifery

f) medical-laboratory diagnostics

g) radiological technology

h) occupational therapy

i) sanitary engineering

j) nursing

k) law

l) administrative studies

m) tax study

n) social work

o) electrical engineering and information technologies

p) computing

6. Year of study:

a) 1.

b) 2.

c) 3.

d) 4.

e) 5.

f) 6.

7. Average grade during the past years of study:

a) 2 – 2.4

b) 2.5-3.4

c) 3.5 – 4.4

d) 4.5 – 5.0

8. Did you pass all the enrolled courses in the previous academic year?

a) Yes

b) No

9. Grade average in the previous academic year 2019/2020.

a) 2 – 2.4

b) 2.5-3.4

c) 3.5 – 4.4.

d) 4.5 – 5.0)

10. Have you re-enrolled a year at university:

a) Yes

b) No

11. If the answer to the previous question is "YES", how many times have you re-entered the year:

a) Only once

b) More than once

12. Do you live in the place of study or outside the place of study?

a) I live in the place of study

b) I live outside the place of study

13. If you answered "I live outside the place of study" to the previous question, please state where you live while studying:

a) I share a room in a student dormitory

b) Rented apartment with colleagues/friends

c) I live alone in an apartment

d) I live with relatives

e) Something else_____________

14. Do you suffer from any chronic diseases?

a) Yes

b) No

15. In the period of one year before the start of the pandemic (until March 2020), did you get sick and were hospitalized as a result?

a) Yes

b) No

16. Have you been diagnosed with any of the psychiatric disorders?

a) Yes

b) No

17. Do you smoke?

a) I don't smoke

b) A few cigarettes a month

c) A few cigarettes a week

d) Up to 10 cigarettes per day

e) Less than a pack of cigarettes per day

f) 1-2 packs of cigarettes per day

g) More than 2 packs of cigarettes per day

Evaluate and mark how the following claims apply to you:

	I don't use	Less than once a month	Once a month	Up to 4 times a month	5 or more times in a month
Persen					
Biorelax					
Vitalia Valeral					
Sereno					
Bonisan					
Relaxerbe					
Cirkulin valeriana					
Melisa					
Pasiflora					
Valeriana					
Marijuana					
Canabis					
Amphetamine					
Ecstasy					
LSD					
Cocaine					
Heroinee					

The following questions refer to the time before the onset of the pandemic and before the earthquake in Zagreb.

1. Before the SARS-CoV-2 virus pandemic, did you ever take tranquilizers?

a) Yes

b) No

2. If the answer to the previous question is "YES", please state what you were taking:

ANXIOLYTICS

a) Alprazolam (Helex, Misar, Xanax)

b) Bromazepam (Lekotam, Lexaurin, Lexillium)

c) Diazepam (Apaurin, Diazepam, Normabel)

d) Oxazepam (Oxazepam, Praxiten)

e) Lorazepam (Lorsilan)

f) Something else ___________________________

HYPNOTICS

g) Midazolam (Dormicum, Midazolam)

h) Nitrazepam (Cerson)

i) Flurazepam (Fluzepam)

j) Something else ____________________________

k) ANTIDEPRESSANTS

3. If you took the above-mentioned medicines, please answer how you got them?

a) via prescription

b) I took it from others

c) I got it myself

d) other ¬____________________

4. How did you take the above medicines?

a) symptomatic, only if necessary

b) I have been prescribed therapy for a certain period of time

c) other __________________

5. How often were the mentioned medicines taken?

a) Everyday

b) Up to 5 times a week

c) Up to 5 times a month

d) Several times a year

The following questions refer to the circumstances since the outbreak of the SARS-CoV-2 virus epidemic and the earthquake in Zagreb.

1. Were you in Zagreb or its surroundings during the earthquake on March 22, 2020?

a) Yes

b) No

2. Have you been prescribed a measure of self-isolation due to the pandemic caused by SARS-CoV-2, and how many times?

a) No

b) Yes, __ times

3. Have you recovered from SARS-CoV-2 infection?

a) Yes

b) No

4. Have you been hospitalized due to SARS-CoV-2 infection?

a) Yes

b) No

5. Evaluate and mark how the following claims apply to you:

	The statement does NOT apply to me at all.	The statement mostly doest NOT apply to me.	Part of the statement applies to me.	Most of the statement applies to me.	The statement applies entirely to me.
The earthquake had a huge impact on my life					

The pandemic and the earthquake have significantly changed my income and funding sources.					
The pandemic and the earthquake reduced the quality of my studies (e.g. poor concentration and poorer learning, poorer sleep, lack of adjustment or poorer conditions for online classes).					
I had difficulties for adjusting to online classes.					
I had technical difficulties in following online classes.					
In addition to the pandemic and the earthquake, and independently of them, I had other aggravating circumstances or difficulties that affected my emotional state.					
I am concerned about the possible impact of contracting this virus on my health.					

I am concerned about the possible impact of contracting this virus on the health of my loved ones.					
I am concerned about the economic consequences of the pandemic.					
I am satisfied with the measures adopted to fight the pandemic.					
I am disturbed by reports about the number of infected and dead people due to the pandemic.					
I am worried about what will happen to academic year 2020/2021					
I am worried about how the pandemic will affect my employment in the future.					
I am worried about how the adaptation of classes to the conditions of the epidemic will affect					

my competence to perform tasks in the future workplace.					

The following questions refer to the time after the onset of the pandemic and after the earthquake in Zagreb

1. Have you ever taken tranquilizers after the beginning of the SARS-CoV-2 virus pandemic?

a) Yes

b) No

2. If the answer to the previous question is "YES", please state what you were taking:

ANXIOLYTICS

a) Alprazolam (Helex, Misar, Xanax)

b) Bromazepam (Lekotam, Lexaurin, Lexillium)

c) Diazepam (Apaurin, Diazepam, Normabel)

d) Oxazepam (Oxazepam, Praxiten)

e) Lorazepam (Lorsilan)

f) Something else ___________________________

HYPNOTICS

g) Midazolam (Dormicum, Midazolam)

h) Nitrazepam (Cerson)

i) Flurazepam (Fluzepam)

j) Something else _____________________________

k) ANTIDEPRESSANTS ___________________________

3. If you took the above-mentioned medicines, please answer how you got them?

a) via prescription

b) I took it from others

c) I got it myself

d) other ___________________

4. How did you take the above medicines?

a) symptomatic, only if necessary

b) I have been prescribed therapy for a certain period of time

c) other _________________

5. How often were the mentioned medicines taken?

e) Everyday

f) Up to 5 times a week

g) Up to 5 times a month

h) Several times a year

6. Have you stopped taking the medicine/s or are you still taking it?

a) I used to take it only when needed and I don't take it anymore

b) I still take it as needed

c) I took it regularly for several weeks

d) other________________

7. Are you aware of the harmful effects of long-term and irrational use of the above-mentioned drugs (tolerance and need for higher doses, addiction...)?

a) Yes, and I carefully use tranquilizers.

b) Yes, but I don't pay much attention to it.

c) I am not aware
