# Supplementary material for: Use of sedative pharmacological agents among biomedical students during the coronavirus disease 2019 pandemic: a cross-sectional pilot study
Source: Croat Med J. 2022 Dec;63(6):570–7. doi: 10.3325/cmj.2022.63.570 (PMC9837717; doi:10.3325/cmj.2022.63.570)
Supplement: Supplementary Table 1 [file CroatMedJ_63_s002.pdf]

Supplementary Table 1. Post hoc analysis (Tukey´s HSD) of the connection between the non-biomedical and subgroups of the biomedical group and the use of SPA before the onset of the pandemic

**P value**	Medicine	Dental medicine	Pharmacy and Medical Biochemistry	Other biomedical fields	Other programs
Medicine	-	.010	.031	.932	.051
Dental medicine	.010	-	1	.352	.634
Pharmacy and Medical Biochemistry	.031	1	-	.440	.774
Other biomedical fields	.932	.352	.440	-	.814
Other programs	.051	.634	.774	.814	-
