# Supplementary material for: Use of sedative pharmacological agents among biomedical students during the coronavirus disease 2019 pandemic: a cross-sectional pilot study
Source: Croat Med J. 2022 Dec;63(6):570–7. doi: 10.3325/cmj.2022.63.570 (PMC9837717; doi:10.3325/cmj.2022.63.570)
Supplement: Supplementary Table 2 [file CroatMedJ_63_s003.pdf]

Supplementary Table 2. Post hoc analysis (Tukey´s HSD) of the connection between the non-biomedical and subgroups of the biomedical group and the use of SPA after the onset of the pandemic

**P value**	Medicine	Dental medicine	Pharmacy and Medical Biochemistry	Other biomedical fields	Other programs
Medicine	-	.026	.066	.844	.039
Dental medicine	.026	-	1	.645	.885
Pharmacy and Medical Biochemistry	.066	1	-	.720	.942
Other biomedical fields	.844	.645	.720	-	.900
Other programs	.039	.885	.942	.900	-
