# Supplementary material for: Use of sedative pharmacological agents among biomedical students during the coronavirus disease 2019 pandemic: a cross-sectional pilot study
Source: Croat Med J. 2022 Dec;63(6):570–7. doi: 10.3325/cmj.2022.63.570 (PMC9837717; doi:10.3325/cmj.2022.63.570)
Supplement: Supplementary Table 3 [file CroatMedJ_63_s004.pdf]

Supplementary Table 3. Method of purchasing SPA before and after the onset of a pandemic.

Method of purchasing SPA	Before the onset of pandemic	After the onset of pandemic
On prescription	85 (6.1%)	73 (5.2%)
Taken from others	76 (5.4%)	58 (4.1%)
Purchased it myself	32 (2.3%)	29 (2.1%)
Other	29 (2.1%)	22 (1.6%)
Non-medical use, i.e., without prescription (A+B+C)	137 (9.8%)	109 (7.8%)
Didn't use	1181 (84.2%)	1221 (87.0%)
