# Supplementary material for: Use of sedative pharmacological agents among biomedical students during the coronavirus disease 2019 pandemic: a cross-sectional pilot study
Source: Croat Med J. 2022 Dec;63(6):570–7. doi: 10.3325/cmj.2022.63.570 (PMC9837717; doi:10.3325/cmj.2022.63.570)
Supplement: Supplementary Table 4 [file CroatMedJ_63_s005.pdf]

Supplementary Table 4. Mode of taking SPA before and after the onset of a pandemic and earthquake

Mode of taking SPA	Before the onset of pandemic	After the onset of pandemic
Symptomatically, only as needed	175 (12.5%)	147 (10.5%)
Prescribed therapy at a certain time	26 (1.9%)	19 (1.4%)
Other	21 (1.5%)	16 (1.1%)
Didn't use	1181 (84.2%)	1221 (87.0%)
