# Supplementary material for: Use of sedative pharmacological agents among biomedical students during the coronavirus disease 2019 pandemic: a cross-sectional pilot study
Source: Croat Med J. 2022 Dec;63(6):570–7. doi: 10.3325/cmj.2022.63.570 (PMC9837717; doi:10.3325/cmj.2022.63.570)
Supplement: Supplementary Table 5 [file CroatMedJ_63_s006.pdf]

Supplementary table 5. Descriptive data for the impact of pandemic and earthquake on lives of students. (N = 1403).

Claim	Mean	Standard deviation	P
The earthquake had a significant impact on my life.	1.82	1.08	<0.001
Pandemic and earthquake have significantly changed my sources of funding.	1.87	1.16	<0.001
The pandemic and earthquake reduced the quality of my schooling.	3.37	1.36	<0.001
I had a hard time adjusting to online lectures.	2.63	1.27	<0.001
I had technical difficulties in following online lectures.	2.10	1.17	<0.001
Regardless of pandemic and earthquake, I had other aggravating circumstances that affected my emotional state.	2.55	1.46	<0.001
I am concerned about the possible impact of this virus on my health.	2.22	1.26	<0.001
I am concerned about the possible impact of this virus on the health of my loved ones.	3.86	1.25	<0.001
I am worried about the economic consequences of the pandemic.	3.55	1.23	<0.001
I am satisfied with the measures taken to combat the pandemic.	2.16	1.06	<0.001
I am worried about reports of the number of infected and dead.	2.39	1.23	<0.001
I'm worried about what will happen with the academic year 2020/2021.	3.14	1.43	<0.001
I am worried about how the pandemic will affect my employment in the future.	3.07	1.47	<0.001
I am concerned that adapting teaching to pandemic conditions will affect my	3.29	1.49	<0.001

competence in the future workplace.			

Values 1-5 replace the following statements: 1 denotes *completely does not apply to me*, 2 *mostly does not apply to me*, 3 *partially applies to me*, 4 *mostly applies to me*, and 5 *fully applies to me*.
