# Supplementary material for: Use of sedative pharmacological agents among biomedical students during the coronavirus disease 2019 pandemic: a cross-sectional pilot study
Source: Croat Med J. 2022 Dec;63(6):570–7. doi: 10.3325/cmj.2022.63.570 (PMC9837717; doi:10.3325/cmj.2022.63.570)
Supplement: Supplementary Table 6 [file CroatMedJ_63_s007.pdf]

Supplementary table 6. Student’s t-test of the relationship between gender and the impact of the pandemic and earthquake on lives of students. N(Female) = 876, N(Male)=527.

Claim	Gender	Mean	Standard deviation	t	P
The earthquake had a significant impact on my life.	Male	1.60	0.93	-6.33	<.001
	Female	1.95	1.15		
Pandemic and earthquake have significantly changed my sources of funding.	Male	1.79	1.11	-1.91	.057
	Female	1.91	1.18		
The pandemic and earthquake reduced the quality of my schooling.	Male	3.21	1.39	-3.39	.001
	Female	3.47	1.34		
I had a hard time adjusting to online lectures.	Male	2.55	1.34	-1.84	.066
	Female	2.68	1.23		
I had technical difficulties in following online lectures.	Male	1.91	1.16	-4.77	<.001
	Female	2.22	1.16		
Regardless of pandemic and earthquake, I had other aggravating circumstances that affected my emotional state.	Male	2.18	1.32	-7.66	<.001
	Female	2.77	1.49		
I am concerned about the possible impact of this virus on my health.	Male	1.96	1.21	-6.11	<.001
	Female	2.37	1.27		
I am concerned about the possible impact of this virus on the health of my loved ones.	Male	3.62	1.31	-5.62	<.001
	Female	4.01	1.18		
I am worried about the economic consequences of the pandemic.	Male	3.34	1.28	-4.83	<.001
	Female	3.67	1.18		
I am satisfied with the measures taken to combat the pandemic.	Male	2.12	1.15	-1.09	.274
	Female	2.19	1.00		
	Male	2.04	1.13	-8.70	<.001

I am worried about reports of the number of infected and dead.	Female	2.61	1.24		
I'm worried about what will happen with the academic year 2020/2021.	Male	2.87	1.45	-5.45	<.001
	Female	3.30	1.39		
I am worried about how the pandemic will affect my employment in the future.	Male	2.75	1.44	-6.57	<.001
	Female	3.27	1.46		
I am concerned that adapting teaching to pandemic conditions will affect my competence in the future workplace.	Male	2.81	1.52	-9.35	<.001
	Female	3.57	1.40		

Values 1-5 replace the following statements: 1 denotes *completely does not apply to me*, 2 *mostly does not apply to me*, 3 *partially applies to me*, 4 *mostly applies to me*, and 5 *fully applies to me*.
