# Supplementary material for: Use of sedative pharmacological agents among biomedical students during the coronavirus disease 2019 pandemic: a cross-sectional pilot study
Source: Croat Med J. 2022 Dec;63(6):570–7. doi: 10.3325/cmj.2022.63.570 (PMC9837717; doi:10.3325/cmj.2022.63.570)
Supplement: Supplementary Table 7 [file CroatMedJ_63_s008.pdf]

Supplementary table 7. Student's t-test of the relationship of belonging to the biomedical and non-biomedical group with the impact of the pandemic and earthquake on the lives of students. N(Biomedical)=627, N(Non-biomedical)=776

Claim	Group	Mean	Standard deviation	t	P
The earthquake had a significant impact on my life.	Biomedical	1.96	1.15	4.39	<.001
	Non-biomedical	1.71	1.02		
Pandemic and earthquake have significantly changed my sources of funding.	Biomedical	1.76	1.08	-3.16	.002
	Non-biomedical	1.95	1.21		
The pandemic and earthquake reduced the quality of my schooling.	Biomedical	3.34	1.36	-0.89	.372
	Non-biomedical	3.40	1.37		
I had a hard time adjusting to online lectures.	Biomedical	2.52	1.18	-3.02	.003
	Non-biomedical	2.72	1.34		
I had technical difficulties in following online lectures.	Biomedical	2.06	1.09	-1.34	.181
	Non-biomedical	2.14	1.22		
Regardless of pandemic and earthquake, I had other aggravating circumstances that affected my emotional state.	Biomedical	2.54	1.42	-0.14	.892
	Non-biomedical	2.55	1.49		
I am concerned about the possible impact of this virus on my health.	Biomedical	2.32	1.25	2.77	.006
	Non-biomedical	2.14	1.26		
I am concerned about the possible impact of this virus on the health of my loved ones.	Biomedical	3.96	1.19	2.70	.007
	Non-biomedical	3.78	1.29		
	Biomedical	3.52	1.19	-0.71	.480

I am worried about the economic consequences of the pandemic.	Non-biomedical	3.57	1.27		
I am satisfied with the measures taken to combat the pandemic.	Biomedical	2.35	1.02	5.85	<.001
	Non-biomedical	2.02	1.07		
I am worried about reports of the number of infected and dead.	Biomedical	2.56	1.24	4.40	<.001
	Non-biomedical	2.27	1.20		
I'm worried about what will happen with the academic year 2020/2021.	Biomedical	3.08	1.38	-1.33	.183
	Non-biomedical	3.18	1.47		
I am worried about how the pandemic will affect my employment in the future.	Biomedical	2.93	1.43	-3.42	.001
	Non-biomedical	3.19	1.50		
I am concerned that adapting teaching to pandemic conditions will affect my competence in the future workplace.	Biomedical	3.52	1.42	5.27	<.001
	Non-biomedical	3.10	1.52		

Values 1-5 replace the following statements: 1 denotes *completely does not apply to me*, 2 *mostly does not apply to me*, 3 *partially applies to me*, 4 *mostly applies to me*, and 5 *fully applies to me*.
